# Microfluidic Devices for Heavy Metal Ions Detection: A Review

**DOI:** 10.3390/mi14081520

**Published:** 2023-07-28

**Authors:** Myrto-Kyriaki Filippidou, Stavros Chatzandroulis

**Affiliations:** Institute of Nanoscience and Nanotechnology, NCSR “Demokritos”, 15341 Aghia Paraskevi, Greece; m.filippidou@inn.demokritos.gr

**Keywords:** microfluidic devices, lab on chip, heavy metal ions, biosensors, water safety

## Abstract

The contamination of air, water and soil by heavy metal ions is one of the most serious problems plaguing the environment. These metal ions are characterized by a low biodegradability and high chemical stability and can affect humans and animals, causing severe diseases. In addition to the typical analysis methods, i.e., liquid chromatography (LC) or spectrometric methods (i.e., atomic absorption spectroscopy, AAS), there is a need for the development of inexpensive, easy-to-use, sensitive and portable devices for the detection of heavy metal ions at the point of interest. To this direction, microfluidic and lab-on-chip (LOC) devices fabricated with novel materials and scalable microfabrication methods have been proposed as a promising approach to realize such systems. This review focuses on the recent advances of such devices used for the detection of the most important toxic metal ions, namely, lead (Pb), mercury (Hg), arsenic (As), cadmium (Cd) and chromium (Cr) ions. Particular emphasis is given to the materials, the fabrication methods and the detection methods proposed for the realization of such devices in order to provide a complete overview of the existing technology advances as well as the limitations and the challenges that should be addressed in order to improve the commercial uptake of microfluidic and LOC devices in environmental monitoring applications.

## 1. Introduction

The environment and human health are threatened by heavy metal ions, such as lead (Pb), mercury (Hg), arsenic (As), cadmium (Cd) and chromium (Cr), which are present in water, air and soil. Heavy metals ions can be categorized into those required by living organisms in small amounts (e.g., iron, manganese, zinc and copper), which only cause toxic effects in high concentrations, and those (e.g., lead, mercury and cadmium) that are highly toxic even in small amounts [1,2,3,4,5].

These contaminants are entering the aquatic and food chains of humans and animals through both human-related activities and natural phenomena. Some examples that result in the release of heavy metal ions include industry activities, urbanization, mining and metals discharges from natural resources, volcanic activities, soil erosion, rainwater and other physical phenomena [2,3,5,6].

Water is the main element of many processes realized in our every-day life; it is related to food and beverages, personal and facilities hygiene, and animal and human healthcare. According to the CDC, heavy metals like arsenic and lead, among others, can easily contaminate tap water [7]. This class of pollutants is characterized by a high toxicity and a density that is greater than 5 g/cm^3^ (five times higher than water density) and also exhibits a low biodegradability and high chemical stability, resulting in the pollutants staying present in the environment for a long time. Such heavy metals are detrimental for fauna and flora, catastrophic to animal and plant habitats and can eventually be lethal to living organisms, including humans [8,9].

**Figure 1:**
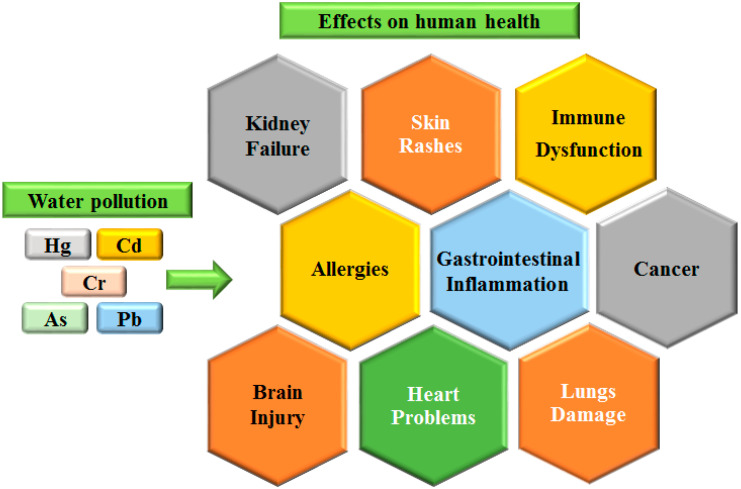
Schematic representation of the effects on human health due to water pollution by heavy metals.

The ways through which heavy metals accumulate into the human body vary and include inhalation from the atmosphere, the ingestion of contaminated food, the drinking of contaminated water and skin contact [10]. Heavy metals are responsible for a plethora of diseases, such as cancer, diseases of the immune system, kidney failure, allergies, heart problems, neurodegenerative diseases, etc. [11,12,13,14]. In Figure 1, the severe impact of water pollution by heavy metals on human health is depicted.

The need for clean and safe water can be addressed with the development of easy-to-use, compact and portable devices targeting heavy metal ions detection. Microfluidic devices offer the possibility to manipulate minute volumes of fluids (typically a few microliters or less) in a way that makes it possible to perform chemical or biological analysis on a single chip. They usually consist of a simple or complex network of microchannels and microchambers, which serve as reaction chambers or reagent reservoirs. When this technology is applied to the detection of heavy metal ions, rapid and affordable devices with reasonable accuracy can be foreseen [15]. LOCs are a subclass of microfluidic devices that strive to include on a single chip all the functions that are necessary to perform a full analysis of a sample in a much the same way as it is performed in a laboratory. In addition, LOCs should preferably operate autonomously and be portable. Thus, LOCs can be considered microlaboratories with many advantages, including a small sample volume, reduced analysis time, low manufacturing cost and great sensitivity [16,17,18,19,20]. Being a relatively new technology, LOCs and microfluidic devices in general are constantly open to new technological approaches, and new materials play an important role in their development. Materials such as glass, silicon, paper, polydimethylsiloxane (PDMS), poly(methyl methacrylate) (PMMA), cyclic olefin copolymer (COC), polyethylene terephthalate (PET), polyvinyl chloride (PVC), polycarbonate (PC) and 3D printing materials are usually used for the fabrication of microfluidics [21]. Silicon and glass were the first-generation materials for microfluidics as they are thoroughly characterized materials with good surface properties and a wide range of well-established processing techniques, while glass has also excellent optical transparency and biocompatibility. Nevertheless, these materials require cleanroom facilities and sophisticated equipment to process, thus rendering the fabrication of LOCs expensive. However, PDMS and thermoplastics, like PMMA, PVC, etc., are commonly used as they are relatively inexpensive and well researched. In particular, PDMS is a material with the following advantages: it is biocompatible, cheap, optically transparent, easy to mold and good for prototyping. Paper microfluidics are characterized by a low cost and can be used to measure desired molecules quickly via visual inspection [22,23].

In addition, LOCs are versatile devices, which can be combined with different detection methods allowing their application in many areas, such as proteomics, gene research, point of care (POC), analytical chemistry, environmental monitoring (heavy metal ions and pesticide detection), food safety [24,25,26,27,28,29,30,31,32,33] and other relevant industrial applications (i.e., liquid–liquid extraction) [34,35].

The fabrication of such innovative microfluidic devices enables the development of portable devices for on-site analysis [36] and can revolutionize the way sciences, such as analytical chemistry and biology, are used. Although conventional spectroscopic techniques, such as atomic absorption spectroscopy (AAS), inductively coupled plasma mass spectroscopy (ICP-MS) or other analytical methods, like high-performance liquid chromatography (HPLC), are currently the gold standard for such analyses due to their high accuracy and sensitivity, they cannot meet the specifications for portability, since most of them require bulky and expensive equipment and highly trained personnel and are time-consuming [4,37].

This review paper is focused on providing a comprehensive literature overview of the most recent and promising examples of microfluidic and LOC devices used for the detection of the most important toxic metal ions, namely, lead, mercury, arsenic, cadmium and chromium. Emphasis is given to the fabrication methods, the materials used, the detection methods proposed and their sensitivity, focusing on highlighting the most promising approaches that already exist in the literature. It is additionally intended to present the limitations of the state-of-the-art examples as well as the challenges in the field. Our study shows that several devices can meet the regulation limits of detection, but few of them have passed to the commercial phase. The reasons for this slow uptake of new detection methods and devices are also discussed at the end of this manuscript.

## 2. Microfluidic and Lab-on-Chip Devices for Heavy Metal Ions Detection Using Various Detection Methods

**Figure 2:**
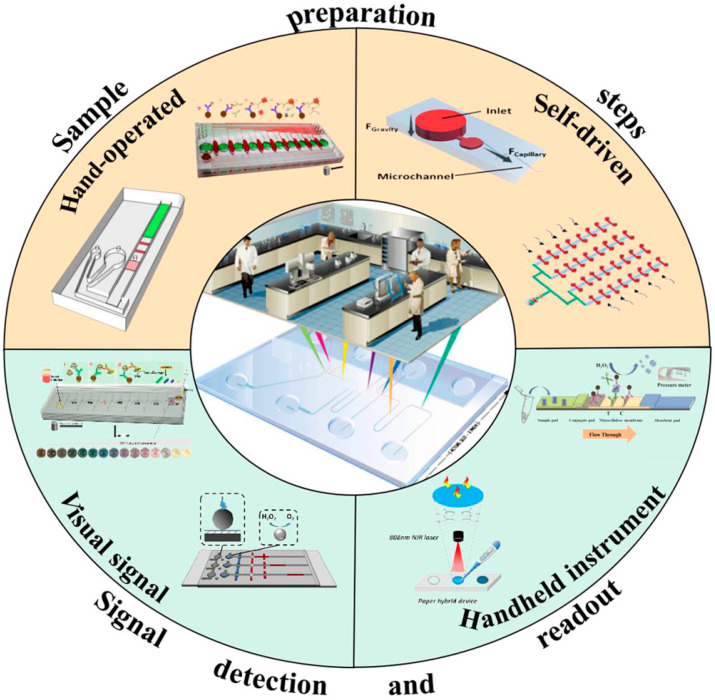
Illustration of miniaturized and integrated microfluidic devices for POC diagnosis. Reproduced with permission from reference [38].

Integrated microfluidic devices enable the transfer of most processes performed on a laboratory bench to a single device, and, in this way, they can meet the requirements of POC systems (Figure 2) [38].

To the question of what drives this extensive research and development of new micro total analysis systems (μTAS), or lab-on-chip devices, the answer is the development of micro- and nanotechnology, new materials, and smaller, smarter and more efficient electronic systems. For example, new nanomaterials offer improved sensing properties, greater sensitivity and greater selectivity. Thus, for the fabrication of innovative LOC devices, special attention must be paid to the choice of the materials used for the fabrication of the sensor that is integrated into the LOC devices. Novel materials, including nanomaterials, are utilized for the sensors’ fabrication for the detection of heavy metals. Some good examples are carbon nanotubes, graphene, reduced graphene oxide and carbon dots (CDs) and polymeric materials (e.g., Polyaniline (PANI), Nafion, Poly(ethylene glycol) (PEG), Poly(vinyl alcohol) (PVA)), as well as metal nanoparticles (e.g., gold, silver, copper) and metal oxides (e.g., CuO, MgO, ZnO, MnO_2_, Fe_3_O_4_, SNO_2_, TiO_2_ and ZrO_2_) [3,6,39,40,41]. Another common approach is to combine bioreceptors with some of the aforementioned materials for the fabrication of novel sensors in which the detection mechanism is related to the biological molecules’ type (e.g., enzymes, antibodies, DNA, RNA, etc.), resulting in the fabrication of immunosensors and aptamer-based and enzyme-based sensors [5]. The development of such nanomaterials with improved electrical and mechanical properties and the development and innovation in the area of microfabrication can transform LOC devices into powerful devices [42].

**Figure 3:**
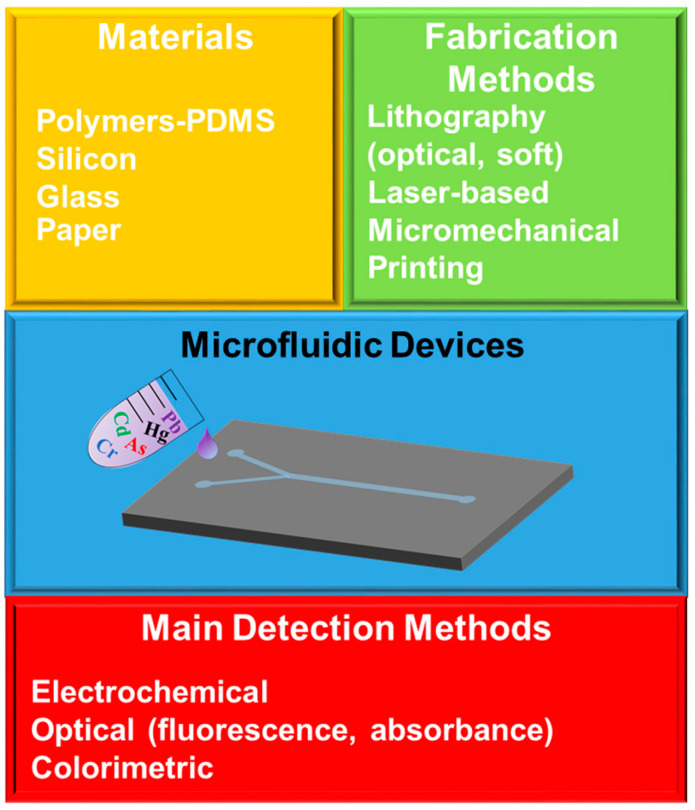
Schematic representation of the materials and the fabrication and detection methods that are used for the realization of microfluidic devices intended for heavy metal ions detection.

At the same time, the selected detection scheme is a very important part of the integrated lab-on-chip or microfluidic device. The main goal is to develop portable, low-cost and easy-to-fabricate and -operate devices that can be used in the point of interest [43], and this is the reason for the rise in alternative detection methods. To this end, several detection methods have been proposed to be used in combination with microfluidics in order to provide portable detection devices. Examples of the sensors that have been proposed for the detection of heavy metal ions are the following: electrochemical, fluorescence, colorimetric, (electro)chemiluminescence, piezoresistive, surface plasmon resonance (SPR) and surface-enhanced Raman scattering (SERS) sensors [15,44,45,46,47,48]. Figure 3 shows a schematic representation of the different materials and fabrication and detection methods that can be combined for the fabrication of microfluidic devices.

Electrochemical sensors [15,44,45] offer high sensitivity, a low limit of detection (LOD) and real-time detection results. Such sensors are ideal for water safety applications, since water pollutants monitoring can be achieved via changes in the electrical response, as the pollutants affect the electrical signal [46]. The most popular electrochemical methods combined with microfluidic devices are based on amperometry, conductometry, potentiometry and voltammetry. Electrochemical sensors also include resistive sensors, in which the response is based on resistance changes or differences, and capacitive-type sensors, in which the device capacity is measured. Furthermore, electrochemical impedance spectroscopy (EIS) is also used for heavy metals ions detection since it is a simple, rapid and low-cost technique. One of the methods that has been widely used for the fabrication of electrochemical sensors intended for environmental monitoring is screen-printing, as reported in the literature [49,50].

Optical methods, like fluorescence, colorimetric methods and surface-enhanced Raman scattering (SERS) spectroscopy, are also very common for heavy metals detection as they have a relatively good sensitivity and specificity and they can be easily combined with microfluidic devices to enable devices suitable for POC applications. Optical methods based on fluorescence, although they are rapid, sensitive, non-destructive and can be employed for in situ or in-line monitoring, usually require more sophisticated external equipment. This equipment used to be bulky and less portable, but nowadays several portable fluorescence sensors exist [51]. Colorimetric methods, however, are usually combined with microfluidic paper-based devices used in water safety for heavy metals detection. Such methods are related to the change in color in the presence of the analyte. In this way, colorimetric methods facilitate the direct detection of heavy metals (liquids or solids) by the naked eye, whereas an optical system can be used for further analysis. This method offers qualitative results in a simple and facile way. Again, in order to achieve the quantification of the results and reach a low LOD, coupling with an image analysis software or device is required, which is nowadays trivial through the wide use of smart portable devices (i.e., mobile phones) [15,47].

SERS is used for the detection of targets on the single-molecule level on the surface using noble metal (e.g., Au, Ag and Cu) nanostructures. SERS is characterized by a high sensitivity and selectivity, less sample pretreatment, a simple operation, a short response time and rich spectral fingerprint information [15,44,52]. Another optical method used for heavy metals detection is SPR [43,53]. SPR has a simple design and high sensitivity, is label free, offers a real-time response and is of low cost.

The rapid growth of chemiluminescence (CL) techniques in the last decades has also led to their use in the detection of different analyte targets including heavy metals amongst others. CL is similar to the fluorescent method and is characterized by high sensitivity. In addition, CL requires simple instrumentation when compared to other optical detection methods, but is restricted by the reagents required (i.e., luminol and ruthenium complexes) and is influenced by the solution composition, its pH and temperature, which can affect reproducibility.

Finally, in some cases microfluidic devices are integrated with piezoresistive sensors in order to fabricate a portable device for rapid heavy metals detection. This kind of sensor relies on a change in electrical resistance under mechanical strain [54]. In the following sections, an overview of these efforts is presented for each one of the most important heavy metal ions.

### 2.1. Lead (Pb) Detection

Lead (Pb) is one of the most dangerous heavy metals that can be found in the environment. It is released by chemical pollution and industrial activities, whereas pollution in drinking water is usually caused by plumbing materials. Some of the negative effects of Pb on humans are that it can act on the nervous system and on skeletal development and it can cause anemia, hypertension, obesity, arrhythmia, kidney failure and even immune system dysfunctions. The fact that even in small amounts Pb^2+^ can inflict damage on adults and children, infants and fetuses especially, stresses the need for Pb^2+^ detection [44,46]. Several examples have been reported in the literature for microfluidic devices combined with optical and electrochemical detection systems for the detection of Pb.

For example, K. A. Shaikh et al. [55] have developed a simple and low-cost LOC system, which incorporates channels, reaction chambers, sensors and actuators for Pb^2+^ detection. By using a DNAzyme fluorescent biosensor, they manage to detect Pb^2+^ concentrations from 10 μM to 500 nM. The DNAzyme scheme has also been used in another work for real-time Pb^2+^ detection, by utilizing fluorescent tags on the DNAzyme, which are immobilized inside a PMMA-based microfluidic device [56]. A microfluidic device with a passive mixer and fluorescent molecular sensors based on calixarene systems for selective Pb^2+^ detection in water is presented in [57]. Using a PDMS Y-shape microchannel bonded on a glass substrate via oxygen plasma treatment, they achieved the detection of 5 ppb of Pb^2+^. A portable and power-free PDMS microfluidic device based on 11-mercaptoundecanoic acid-functionalized gold nanoparticles for Pb^2+^ detection is reported in [58]. The detection is achieved using a microscope or a water drop as a magnifier, and 10 μM of Pb^2+^ are detected by this scheme.

**Figure 4:**
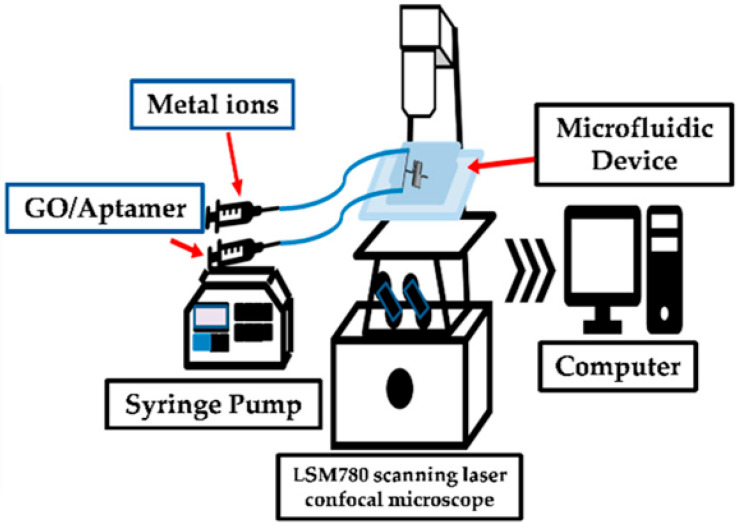
Schematic representation of the measurement system used for the detection of Hg^2+^ (0.70 ppb) and Pb^2+^ (0.53 ppb) inside a PDMS microfluidic device. The GO/aptamer suspension and the metal ion solutions are introduced inside the microfluidic device through the two inlets of the device using a syringe pump. The detection is achieved via the GO/aptamer suspension fluorescence intensity change, while the quenching effect is evaluated via image analysis software installed on an interfaced PC. Reproduced with permission from reference [59]. This article is an open-access article distributed under the terms and conditions of the Creative Commons Attribution (CC BY) license.

W. H. Huang et al. [59] have fabricated a PDMS microfluidic device based on graphene oxide (GO)/aptamer sensors for the simultaneous detection of Hg^2+^ and Pb^2+^ ions. They have achieved the detection of concentrations of around 0.70 ppb and 0.53 ppb for Hg^2+^ and Pb^2+^, respectively, which are lower than those proposed by the World Health Organization (WHO) (Figure 4). An alternative method for Pb^2+^ detection is achieved using a microfluidic analogue of Wheatstone-bridge (SMAW) with a microgel [60]. In particular, the PDMS/glass-based SMAW microchip allows signal conversion and amplification for the real-time continuous detection of Pb^2+^, with a detection limit of 10^−14^ M, utilizing an optical microscope.

Another promising portable and low-cost platform is presented in [61]. In particular, a paper-based microfluidic device for heavy metal ions detection using DNA strands and electrochemiluminescence (ECL) labels was fabricated. In this way, they have achieved the simultaneous detection of 10 pM Pb^2+^ and 0.2 nM Hg^2+^. N. Fakhri et al. [62] have also proposed a paper-based microfluidic for lead detection in water. They have combined aptamers with gold nanoparticles for the colorimetric detection of Pb^2+^, and, using two types of filter paper, namely, Whatman No. 1 and nylon filter papers, they have detected 1.2 nM and 0.7 nM of Pb^2+^, respectively. Furthermore, a microfluidic paper-based analytical device (µPAD) has been proposed in which lead detection is either performed via naked eye estimation of the color change on the pad (principle 1) or using image analysis for the color change intensity (principle 2) [63]. A sodium rhodizonate reagent (NaR) is used in both approaches and after the injection of the sample a color change is observed; while using principle 1, the limit of detection is 0.756 mg L^−1^.

In addition, J. Zhou et al. [64] have fabricated a portable paper-based device for heavy metals detection with quantitative information, resulting in a promising platform for the rapid testing of metal ions. In this approach, ZnSe quantum dots (QDs) are combined with ion imprinted polymers (IIPs) for the detection of Cd^2+^ and Pb^2+^ ions with detection limits of 0.245 μg L^−1^ and of 0.335 μg L^−1^, respectively. Finally, a cloth/paper hybrid microfluidic analytical device with a fluorescence sensing cloth-based component has been proposed for the detection of Hg^2+^ and Pb^2+^ ions with concentrations down to 0.18 and 0.07 μg L^−1^, respectively [65]. For the realization of this fluorescence sensing cloth-based component, which is assembled on a rotary μPAD substrate, first quantum dots are grafted onto the cotton cloth and then IIPs are used for further modification. While using this scheme, they have managed to optimize the portability of the device.

**Figure 5:**
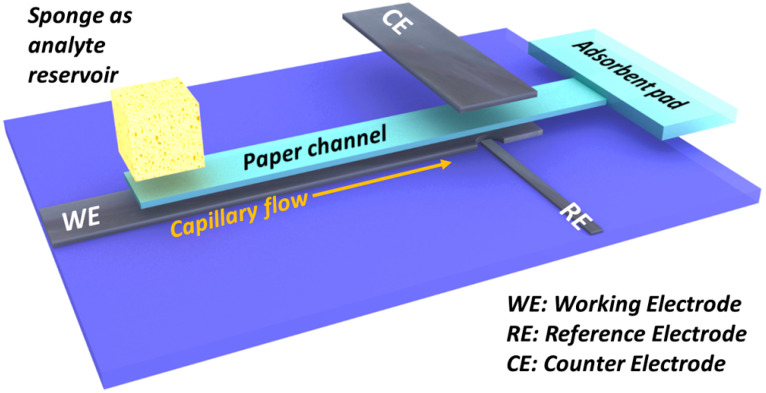
Illustration of a paper-based (light blue) microfluidic device with graphite foil (black) for Cd^2+^ and Pb^2+^ monitoring. The electrodes are placed on a PMMA substrate to facilitate operation. This device with the carbon-based sensor has a three-dimensional layout in which the counter and the working electrodes are facing each other and as a result the microfluidic paper channel is placed among these two electrodes. A piece of sponge is used as analyte reservoir and an absorbent pad is placed on the channel end and capillary flow is enabled. Reproduced with permission from reference [68]. This is an open-access article published under an ACS AuthorChoice License, which permits the copying and redistribution of the article or any adaptations for non-commercial purposes.

Many attempts have also been reported in the literature for paper-based microfluidic devices that are combined with electrochemical detection systems. Such systems can be more versatile and more quantitative. Z. Nie et al. [66] have described a paper-based microfluidic device in which the screen-printed electrodes (SPEs) made from conducting inks, like carbon or Ag/AgCl, are fabricated on paper or polyester film for Pb^2+^ detection. Furthermore, M. Medina-Sánchez et al. [67] have fabricated a portable lab-on-paper device, which also allows sample pretreatment for the electrochemical sensing of lead and cadmium with concentrations down to 7 and 11 ppb, respectively. The device was fabricated using wax and screen-printing technologies. A miniaturized paper-based microfluidic device with a three-dimensional layout with working and counter electrodes using a graphite foil was used for Cd^2+^ and Pb^2+^ detection, and this electrochemical-based microfluidic device can detect concentrations down to 1.2 μg L^−1^ and 1.8 μg L^−1^, respectively (Figure 5) [68]. In another approach, a boron-doped diamond paste electrode, which can be stencil-printed, was combined with paper-based microfluidic devices for the realization of a low-cost, high-performance electrochemical sensor for the detection of Pb^2+^ and Cd^2+^ [69]. The detection limit of this device is 1 and 25 ppb for Pb^2+^ and Cd^2+^, respectively.

**Figure 6:**
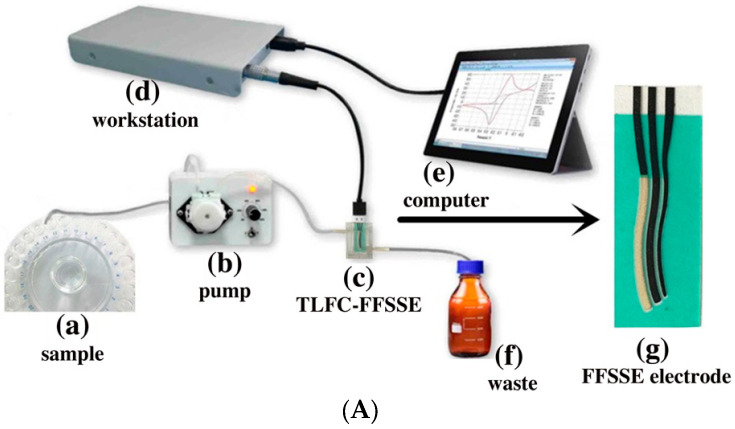

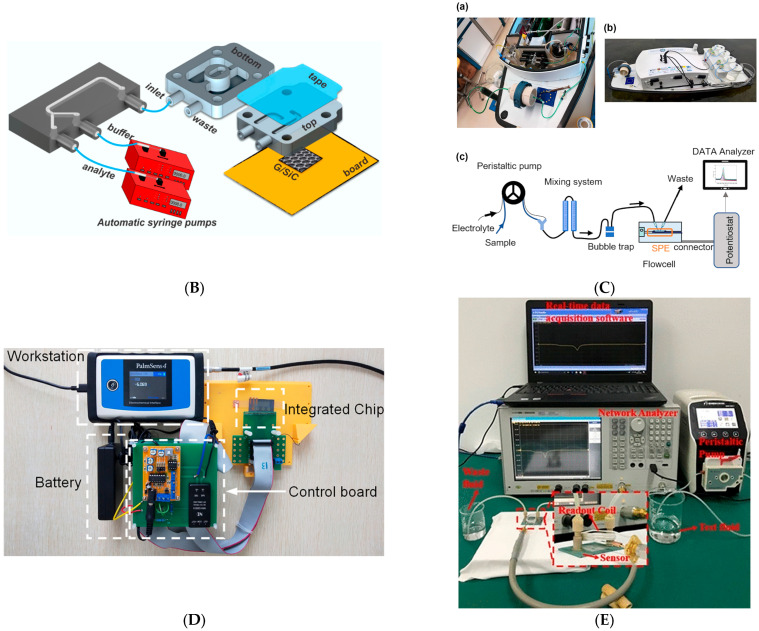
Examples of different microfluidic devices proposed for heavy metal ions detection: (**A**) An electrochemical sensor (FFSSE) combined with a 3D-PTLFC for Pb^2+^ detection. Reproduced with permission from reference [72]. (**B**) Schematic representation of a lab-on-chip device consisting of a 3D-printed microfluidic device and an EG sensor for Pb^2+^ detection. Reproduced with permission from reference [75]. This article is an open-access article distributed under the terms and conditions of the Creative Commons Attribution (CC BY) license. (**C**) (**a**,**b**) Images of an autonomous vehicle for Pb^2+^ detection in surface water and (**c**) illustration of the main parts of the microfluidic device of the particular system. Reproduced with permission from reference [78]. (**D**) The detection system for Pb^2+^ monitoring in tap water using a device comprising of DMF platform and an electrochemical sensor. Reproduced with permission from reference [79]. (**E**) The measurement set up for Pb(NO_3_)_2_ and Cd(NO_3_)_2_ detection using a microfluidic device. Reproduced with permission from reference [81]. This is an open-access article distributed under the terms and conditions of the Creative Commons Attribution CC-BY-NC-ND license.

W. Jung et al. [70] have fabricated a miniaturized polymer lab chip sensor that is reusable, using microfabrication technology for the continuous and on-site detection of heavy metals and especially Pb^2+^. The device consists of a microfluidic channel and a fully integrated sensor with a planar Ag working electrode and Ag counter/quasi-reference. The LOD was measured via square-wave anodic stripping voltammetry (SWASV) and it was found to be 0.55 ppb. A transparent integrated microfluidic device with a 3D-printed thin-layer flow cell (3D-PTLFC) and an S-shaped SPE for heavy metal ion stripping analysis is presented in [71]. This device has a high performance and is characterized by a low cost, and the LOD for Pb^2+^ is 0.3 µg L^−1^. An electrochemical system using 3D-PTLFC and a flow-field shaped solid electrode (FFSSE) was used for Pb^2+^ detection (Figure 6A) [72]. This square-wave anodic stripping voltammetry (ASV) system is characterized by a better sensitivity and reproducibility compared to a traditional ASV-based method with a detection limit of 0.2 μg L^−1^ (0.2 ppb). B. Ding et al. [73] have fabricated a 3D-printed flow reactor in which porous carbon electrodes made via direct laser sintering on polymer films are placed. This scheme is used for Pb^2+^ detection with an LOD of 0.0330 mg L^−1^. A. Chałupniak et al. [74] have fabricated a LOC platform that consists of a screen-printed carbon electrode (SPCE), a PDMS chip and a GO–PDMS chip for the preconcentration and detection of heavy metals like Pb^2+^. Another lab-on-chip device in which a 3D-printed microfluidic device is combined with an epitaxial graphene (EG) sensor was fabricated for Pb^2+^ electrochemical detection [75] (Figure 6B). The authors reported a quite low LOD of 95 nM for Pb^2+^, which is attributed to the high sensitivity of the sensing material. In addition, a portable resistive device for the detection of Pb^2+^ in water, with an LOD of 0.81 nM and a shelf-life of ~45 days, was reported in [76]. For the realization of this device, they combined miniaturized electronics with a microfluidic well, while the sensing material is based on α-MnO_2_/GQD nanocomposites.

Furthermore, J. Dai et al. [77] have reported an integrated and miniaturized microfluidic electrochemical sensor for Pb^2+^ detection. For the fabrication of the device, a “glass-silicon-glass” sandwich structure was developed, while the microsensor has a nanochannel liquid conjunct Ag/AgCl reference electrode, a working electrode with a three-dimensional Au micropillar array and a detection chamber for sample measurement. Finally, the device is characterized by a good sensitivity, repeatability and selectivity and a wide detection range, enabling its use for water quality monitoring, and its LOD is 0.13 μg L^−1^. In another work, a microfluidic device with an electrochemical carbon sensor was used for Pb^2+^ detection, since this kind of sensor decreases the LOD, which is 40 ppt, by three orders compared to traditional heavy metal sensors.

A novel autonomous robotic system for Pb^2+^ detection in surface water has been developed [78]. More specifically, a microfluidic device was combined with an electrochemical sensor made from carbon-based screen-printed electrodes. The device has the ability of performing 39 measurements per day, and the limit of detection for Pb^2+^ for this integrated system is 4 µg L^−1^ (Figure 6C). M. Zhang et al. [79] have proposed a chip consisting of an electrochemical sensor and a digital microfluidic (DMF) platform for the detection of Pb^2+^ in tap water (Figure 6D). This portable device has a low power consumption and relay control modules, it enables automatic sample pretreatment, sensing, waste collection and data acquisition, and the sensing node can be functional for several years using a normal battery. Another example of a microfluidic device with an electrochemical sensor is presented in [80]. In particular, a 3D Ag-rGO-f-Ni(OH)_2_/NF composite is utilized as an amplifier of the electrochemical signals under the synergistic effect of thermocapillary convection, with which an acceleration of the preconcentration process and reduction in the detection time (it saves 300 s of preconcentration time) is achieved. This portable device is controlled by a smartphone and the LOD of Pb^2+^ in river water is 0.00498 μg L^−1^. Finally, a microfluidic channel was combined with a wireless sensor in a low-temperature cofired ceramic substrate among the capacitor plates for the detection of various metals ions, like Pb(NO_3_)_2_ and Cd(NO_3_)_2_ (Figure 6E) [81]. Thus, via the changes in the amplitude of reflection coefficient, the detection of metal ion solutions in a low concentration range of 0–5 mM can be obtained, and the LOD of this kind of sensor can be as low as 5 μM.

### 2.2. Mercury (Hg) Detection

Mercury (Hg) is another harmful pollutant that affects the environment, and it is produced by various industries, such as the paper, pharmaceuticals and horticulture industries. It can be absorbed by fish or shellfish and enter to human body via the food chain or by drinking water, affecting in this way cardiovascular, gastrointestinal, urinary and neurological systems. The toxicity of Hg and its compounds differs according to the method of inhalation or ingestion or the amount. The provisional tolerable weekly intake of Hg in foods proposed by the Food and Agriculture Organization of the United Nations and the WHO is 0.3 mg. No detectable negative effects are caused to the human body below this limit, but this high safety standard stresses the need for accurate Hg detection [44,46].

**Figure 7:**
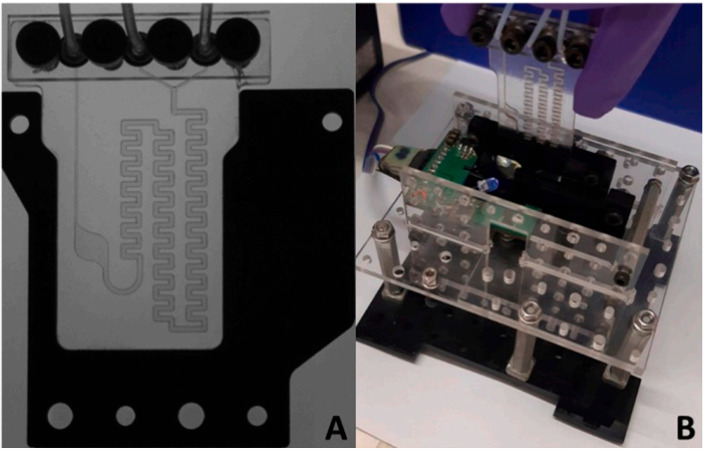
Images of (**A**) the microfluidic device and (**B**) the compact optical-based detection system in which the microfluidic device is placed for heavy metal ions detection, e.g., Hg^2+^ and Pb^2+^. Reproduced with permission from reference [85].

Similarly to Pb^2+^, many efforts have been made for the detection of Hg using microfluidics devices and optical detection systems. For example, Hg^2+^ was detected via an optical microfluidic device with ionophore modified gold nanoparticles [82]. This device, which uses an easy and automated procedure, is selective to Hg^2+^ among different ions that may exist in environmental water samples, with an LOD of 11 ppb. Guilong Peng et al. [83] have achieved the detection of 0.031 μM of Hg^2+^ using a microfluidic chip, fabricated after the bonding of PDMS with a cleaned glass slide via an oxygen plasma treatment step. The chip is then integrated with on-line complexing and laser-induced fluorescence detection. The device is based on a rhodamine derivative (RD), which is the fluorescent chemosensor and thus, by combining the microfluidic technology with the chemosensor, they have developed a portable and low-cost device with less consumption of sample and reagents, high selectivity and a rapid response time, demonstrating the potential in on-site analysis. In addition, a digital microfluidic fluorometric sensor was proposed for Hg^2+^ detection in costal seawater [84]. As previously discussed, a rhodamine-based fluorescent probe is used for the detection of Hg^2+^ due to its fast response at ambient temperatures and high selectivity in Hg^2+^, and by changing the sensitive fluorescent probe other metals like Pb and Cd can also be detected. This rapid and low-cost device enabled the detection of mercury with a concentration down to 1.4 ppb. Another example for the detection of toxic metals is the use of CDs as the selective optical reagents in a microanalyzer consisting of a COC analytical microsystem, a flow management system and a miniaturized customized optical detection system (Figure 7) [85]. By utilizing this device, they have managed to detect various heavy metals ions such as Hg^2+^ and Pb^2+^ with detection limits ranging from 2 to 12 ppb and even lower for Hg^2+^.

Paper-based microfluidics are constantly gaining ground in Hg^2+^ monitoring. An example of this case is presented in [86], in which they have managed to quantify mercury ions using a distance-based detection method on a microfluidic portable and low-cost μPAD. An insoluble colored complex is obtained when mercury ions interact with dithizone, and mercury is quantified by measuring the length of the colored precipitate utilizing a printed ruler along each device. A three-dimensional origami microfluidic paper-based chip for fluorescence detection of Hg^2+^ ions is reported in [87]. In particular, in this device CdTe QDs are combined with ion imprinting technique resulting in a quantitative information and in a detection limit of 0.056 μg L^−1^. In another similar work, a three-dimensional microfluidic paper-based device was presented as a solution for the detection of several metals (i.e., Cd^2+^, Pb^2+^, Hg^2+^) in coastal waters. The design of this device enabled a more homogeneous permeation of the fluid in the paper chip, resulting in a 25-fold enrichment for each metal. The reported LOD ranged from 0.007 to 0.015 μg L^−1^ for all metals tested [88]. K. Patir et al. [89] have used nitrogen-doped carbon dots (NCDs) for fluorescence detection of Hg^2+^. Thus, by fabricating a filter paper-based microfluidic device with NCDs they have achieved detections of Hg^2+^ with the concentration down to 0.1 μM. A chemically functionalized paper-based microfluidic platform, in which silane compounds terminating in amine (NH_2_), carboxyl (COOH) and thiol (SH) are immobilized on a patterned chromatography paper, has been developed for the detection of various heavy metal ions, like Cr (VI) and Hg^2+^ [90]. For the quantification of the heavy metals detection, they used color intensity measurements, and the LODs for Cr (VI) and Hg^2+^ are 0.18 ppm and 0.19 ppm, respectively. Another paper-based device, in which a smartphone is used as a colorimetric analyzer for monitoring Hg^2+^ in water samples, enabling use by unskilled users, is described in [91]. Using this device with unmodified silver nanoparticles (AgNPs) on the detection zones, an LOD down to Hg^2+^ 0.003 mg L^−1^ is achieved. G. Dindorkar et al. [92] have also considered that the fabrication of a device for mercury detection is an imperative need. So, they have fabricated a µPAD, which is combined with a simple colorimetric android-based application, for Hg^2+^ quantification with concentrations from 0.1g L^−1^ to 0.001 mg L^−1^ in water. The detection scheme proposed included gold nanoparticles (AuNPs) functionalized with Papain and 2,6-pyridinedicarboxylic acid. A novel μPAD device, which is combined with a colorimetric sensor, was proposed for the detection of Hg^2+^ ions in aqueous solutions [93]. In particular, cysteamine@gold nanoparticles (CysA@AuNPs) are used with amino acids as ion detection probes and Hg^2+^ was detected via a naked eye color change for concentrations down to 0.001 ppm. In another example, a paper-based analytical device utilizing colorimetry for the quantification of various metal ions such as Hg^2+^ was also presented [94]. The metals are monitored via the reaction between the metal ions and complexing agents in the detection zone and the LOD of the proposed device for Hg^2+^ is 0.20 mg L^−1^. In another approach, a paper disc device, which works with a compact smartphone-based reading device, was proposed for the detection of water contaminants, such as Hg^2+^ and Pb^2+^ [95]. The method is based on fluorescence, and concentrations down to 20 nM and 4 nM for Hg^2+^ and Pb^2+^, respectively, were detected.

A novel label-free device without the requirement of bulky equipment, enabling portability, was reported in [96]. In particular, an SERS substrate in a PDMS microfluidic channel, using silver nanostructures inside the microfluidic channel, for the rapid detection of Hg ions compared to conventional SERS devices, was fabricated. This device can detect Hg ions in aqueous solutions with a high sensitivity and good selectivity, and the reported LOD is 1 × 10^−7^ M. W. Zhang et al. [97] have also achieved the detection of Hg^2+^ using a localized surface plasmon resonance (LSPR) nanosensor and a microfluidic chip made from PDMS, which is bonded with a cover glass. This device, with nanostructures formed by nanorods, can monitor Hg^2+^ in real-life water samples with an LOD of 2.7 pM.

Microfabrication technology for the fabrication of an on-chip integrated electrochemical detector with planar electrodes (Au–Ag–Au three-electrode system) and a PDMS microfluidic channel was utilized in reference [98]. In this way, a simple in use, low analyte consumption, rapid response, miniaturized device with a high sensitivity and good reproducibility is developed for Hg^2+^ monitoring. This device has an LOD of 3 ppb and shows great potential for the application of in situ or on-line mercury detection. H. L. Nguyen et al. [99] have also combined a PDMS microfluidic chip with an electrochemical sensor for mercury detection. They have used a complex of PANI and sodium dodecyl sulfate to modify the electrode placed inside the microfluidic chip and the device limit of detection was reported to be as low as 2.4 nM.

In another approach, microfluidic technology was again coupled with electrochemical detection [100]. The reported device consisted of an array of interdigitated microelectrodes with a small volume (~4 μL) and the authors studied the effect of the spacing between the electrodes. The LOD for Hg^2+^ was 12.4 ± 1.95 μg L^−1^, which is nevertheless higher than the LOD requirements of the US Environmental Protection Agency (EPA) (2 μg L^−1^).

Finally, a microfluidic device with unmodified ITO electrodes, which were formed using a CO_2_ laser ablation method, has been presented for mercury detection [101]. The microchannel was made from PDMS and it was bonded via plasma with a glass containing the ITO electrodes. The microfluidic device has an integrated pump for the analytes’ introduction and a portable potentiostat connected with a smartphone for various metals’ detection (i.e., mercury). The LOD for mercury was reported to be 3.19 µM, and it was reported that simultaneous detection of various metals such as copper and mercury was also possible.

### 2.3. Arsenic (As) Detection

Another toxic metal that can easily pollute drinking water via agrochemical and industrial waste is arsenic (As), which is reported as the most toxic substance among others. This heavy metal can harm multiple organ systems in the human body, like the bladder, liver, lungs and skin, and chronic arsenic exposure via drinking water is the reason for anemia and thrombocytopenia, blackfoot disease, dyspepsia, peripheral neuropathy, pigmentation and keratosis, and leucopenia. The WHO and the EPA have set the maximum contaminant level of As in drinking water at 10 ppb. Since the arsenic compounds are highly toxic, there is an urgent need for their detection. Some of the microfluidic devices used for this purpose are described below [44,46,102].

P. Nath et al. [103] have described the detection of As^3+^ ions using a paper-based microfluidic, realizing a portable, power-free chip device. The device is based on a gold nanosensor (Au–TA–TG) for the detection of 1.0 ppb; according to the WHO, this value is lower than the reference standard for drinking water. Similarly, a μPAD with a gold nanosensor, which was functionalized with α-lipoic acid and thioguanine (Au–TA–TG), was proposed for the monitoring of arsenic in groundwater [104]. Authors have proved that this device is able to detect if the arsenic level is above or below the WHO guideline level of 10 μg L^−1^. M. Gabhane et al. [105] succeed in detecting 5 ppb of As^3+^ ions using a combination of gold nanoparticles and a paper-based microfluidic device equipped with a mini microscope for colorimetric monitoring.

**Figure 8:**
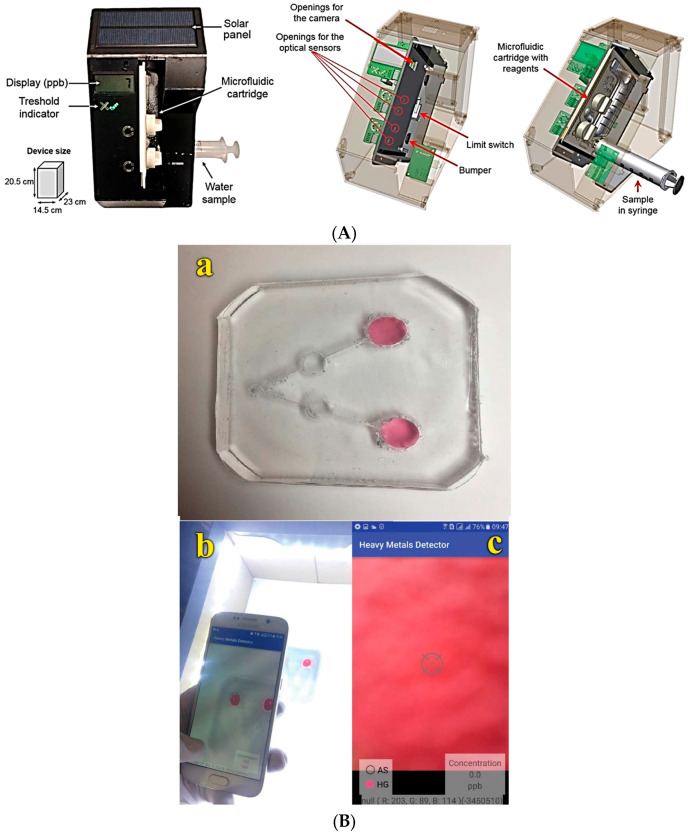
(**A**) Images of the portable colorimetric-based device for arsenic detection in drinking water and image of the detection device from the 3D CAD, where the disposable microfluidic cartridge and the way that the water sample is inserted are depicted. Reproduced with permission from reference [106]. (**B**) Images (**a**) of the portable PDMS microfluidic kit, (**b**) of the way that the measurement is realized using a smartphone and (**c**) of the application utilized for the detection of heavy metals, such as arsenic and mercury. Reproduced with permission from reference [108]. This article is distributed under an Attribution-NonCommercial 3.0 license (CC BY-NC 3.0).

In another interesting work, a semi-automated portable device with a disposable microfluidic cartridge for untrained users was presented for the detection of arsenic (As^3+^ and As^5+^), and with this colorimetric device they have measured concentrations in drinking water down to 1 μg L^−1^, which is again lower than the WHO guidelines of 10 μg L^−1^ (Figure 8A) [106]. In another work, As^3+^ ions are detected in a continuous flow by surface-enhanced Raman scattering on a zigzag PDMS microfluidic chip [107]. For this purpose, they have utilized AgNPs, for the SERS enhancement substrate, conjugated with glutathione with 4-mercaptopyridine, and the highly sensitive and reproducible analysis of the device is performed very fast (several minutes). This kind of device, with an LOD of 0.67 ppb, has great potential in use with a lab-on-chip technique for environmental monitoring. Finally, a portable smartphone-based PDMS microfluidic kit has been developed for the detection of various heavy metals like arsenic and mercury (Figure 8B) [108]. In this colorimetric based device used for POC detection, the response is obtained through the formation of nanoparticle aggregates via the interaction of gold nanoparticles with dithiothreitol—10,12-pentacosadiynoic acid and lysine in the presence of As^3+^ and Hg^2+^ ions, enabling the monitoring of water sample concentrations in ranges from 710 to 1278 μg L^−1^ and from 10.77 to 53.86 μg L^−1^ for As^3+^ and Hg^2+^ ions, respectively.

**Figure 9:**
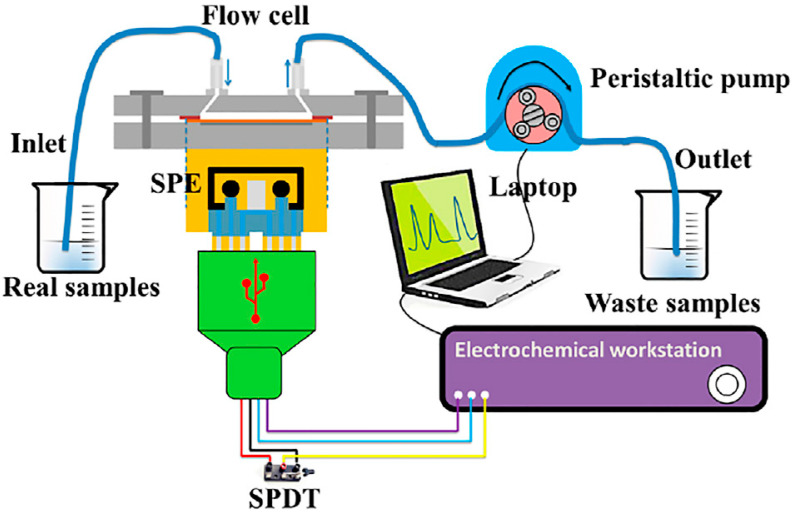
Schematic representation of the flow injection system for various heavy metals’ detection in water, e.g., As^3+^, Pb^2+^ and Cd^2+^. The 3D-printed polymer flow cell with an integrated electrochemical sensor is depicted. Reproduced with permission from reference [113]. This is an open-access article distributed under the terms of the Creative Commons Attribution License (CC BY).

Subsequently, several examples in which a microfluidic channel is combined with an electrochemical sensor for heavy metal ions detection have also been reported. For example, U. Kim et al. [109], knowing the effects of arsenic contaminated drinking water, have decided to fabricate a microfluidic device with an electrochemical sensor combined with a handheld electrochemical analyzer for rapid detection at the test site. This low-cost and portable point-of-use microfluidic platform is comprised of a plastic substrate based on polyphenylene ether above which electrochemical sensors are ink printed (carbon, silver and silver/silver chloride ink electrodes). In another example, a microfluidic device that had a PDMS reaction chamber and single-walled carbon nanotubes patterned on a glass substrate coupled with gold nanoparticles at specific positions inside the microfluidic channels has been reported [110]. The device exhibited a high sensitivity and can detect 30 ppb of As^3+^ in 60 s. In addition, a microfluidic device with an electrochemical sensor based on a gold thin-film electrode modified with AuNPs has been presented in [111]. Using the aforementioned device, As^3+^ with a concentration 0.42 μg L^−1^, lower than WHO standards (10 μg L^−1^), can be detected in water. Furthermore, a point-of-use microfluidic device consisting of radial channels and an electrochemical sensor for arsenic monitoring in water samples was reported in reference [112]. The authors achieved nanoelectrokinetic preconcentration of the target via ion concentration polarization and managed to preconcentrate and detect As^3+^ with concentrations as low as 1 ppb without including valves. Finally, G. Zhao et al. [113] have integrated electrochemical sensors in a 3D-printed polymer flow cell for various heavy metals’ detection in water, such as As^3+^, Pb^2+^ and Cd^2+^ (Figure 9). A flexible substrate is used for the fabrication of the sensor, which consists of screen-printed commercial graphite ink (working and counter electrode) and a Ag/AgCl ink (reference electrode) for the realization of SPEs on the polyimide substrate. By modifying the two different working electrodes with a (BiO)_2_CO_3_-reduced graphene oxide–Nafion nanocomposite, the detection of 0.8 μg L^−1^ Cd^2+^ and 1.2 μg L^−1^ Pb^2+^ is achieved. With an Fe_3_O_4_ magnetic nanoparticle–Au nanoparticle–ionic liquid nanocomposite, 2.4 μg L^−1^ of As^3+^ is detected.

### 2.4. Cadmium (Cd) Detection

Minerals include metals like cadmium (Cd) in the form of chemical compounds. Such compounds are utilized in industries like electroplating, solar cells, battery and plastic stabilizers [44,46]. In addition, they are commonly discharged into the environment via waste gas and waste water. Such substances are toxic since they can enter the human body through the food chain and affect the kidneys and liver, resulting in chronic poisoning. The toxicity limit for humans is 100 mg. Below, some of the microfluidic devices used for this purpose are presented.

**Figure 10:**
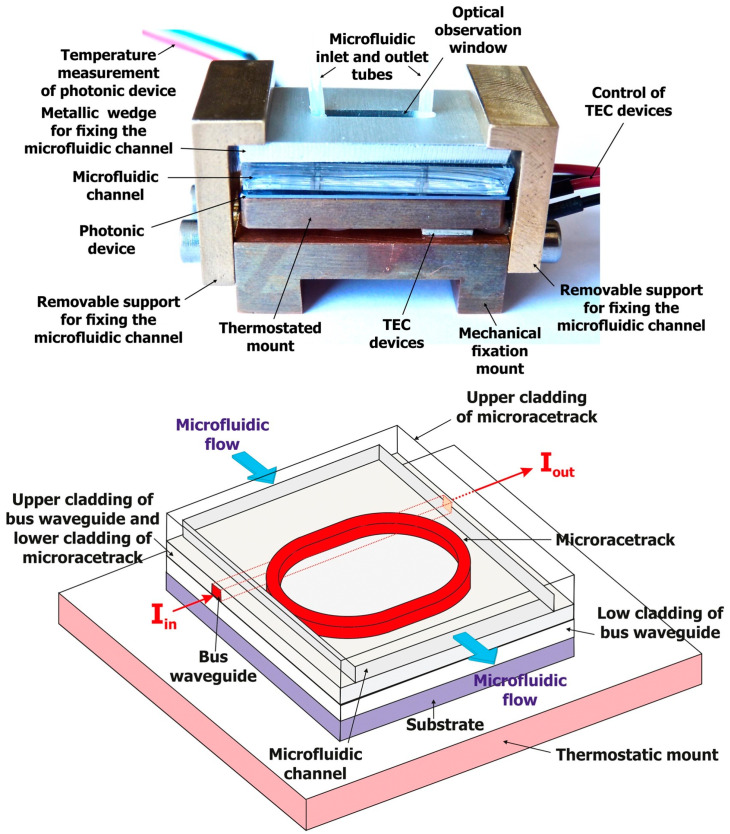
(**Top**) Image of an optofluidic sensor device for Cd detection in tap water. The optical device and the microfluidic chamber are bonded together by applying pressure onto the flexible microfluidic chamber. The optofluidic cell is installed on a thermostatic mount to control the sensor temperature, since the response of the polymeric microresonator is thermally dependent, whereas thermo-electric cooling (TEC) devices are used for temperature stability. The above components are finally placed onto a mechanical mount for the insertion of the sensor in the measurement bench. (**Bottom**) Schematic representation of the optical detection, label-free device. The optofluidic sensor has a SU-8 microracetrack vertically bonded to a SU-8 bus waveguide, which acts as an input/output channel. The lower and the upper cladding of the bus waveguide are made of a thick silica layer, which is deposited on a silicon substrate, and a CYTOP amorphous fluoro-copolymer, respectively. The microfluidic channel is molded inside a polymer substrate, which is afterwards integrated upon the lower cladding of the optical microracetrack, while the direction of the microfluidic flow is perpendicular to that of the bus waveguide. Reproduced with permission from reference [116].

For example, H. Zhang et al. [114] have integrated a fluorescent sensor based on Rhod-5N in a microfluidic detection system comprising a Y-shape PDMS microchannel fixed on a glass substrate. Using this device, 0.45 μg L^−1^ of Cd^2+^ can be detected, and solid-phase adsorption on aminopropyl silica beads is used in order to avoid Pb^2+^ interference. In another work, a microfluidic device for the selective and sensitive monitoring of different heavy metals, like As^3+^, Cd^2+^ and Pb^2+^, was presented [115]. In particular, the device was made after bonding PDMS with a glass substrate, which incorporated a solid-phase extraction unit for sample pretreatment, a micropump and a micromixer for flow control and a detachable graphene oxide quantum dot (GOQD) as a sensing array. For the fluorescence detection of heavy metal ions, the GOQD of the sensor array is linked with DNA aptamer. Using this scheme, As^3+^, Cd^2+^ and Pb^2+^ can be detected with detection limits of 5.03 nM, 41.1 nM and 4.44 nM, respectively. In another approach, an optofluidic sensor for Cd^2+^ detection in tap water has been developed (Figure 10) [116]. This device enables label-free detection of Cd^2+^ and has a polymeric microresonator functionalized with a cadmium ion-specific ligand, and a PDMS microfluidic channel is used for the introduction as well as for the flow control of the analyte solution, forcing it to pass onto the microresonator, which was placed at the center of the channel. This group, using the particular device, has achieved the detection of the presence of Cd^2+^ with concentrations down to 0.36 ± 0.10 nM or 38.9 ± 10.6 ng L^−1^ in tap water, whereas when a post-measurement median filter was used the LOD was 25 times lower (0.17 ± 0.04 nM or 18.3 ± 4.4 ng L^−1^) than the European maximum acceptable concentration in drinking water (0.45 μg L^−1^).

**Figure 11:**
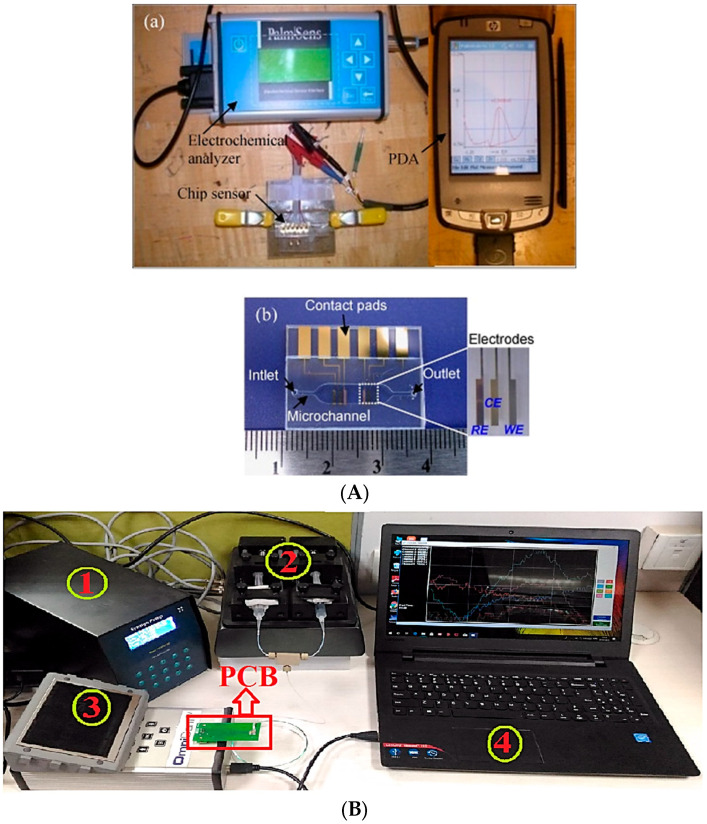
(**A**) Images (**a**) of the experimental set up for on-site measurement of Cd^2+^ and (**b**) of the disposable polymer lab chip with the microchannel and the integrated electrochemical sensor. Reproduced with permission from reference [118]. (**B**) Image of the portable experimental set up for Cd^2+^ detection in ground water using a microfluidic platform consisting of a microcantilever-based piezoresistive sensor. (1 and 2) are used for the flow control (syringe pump and controller), (3) the piezoresistive module and (4) the interface software running on a PC. Reproduced with permission from reference [123]. This article is published under a CC BY license.

An on-site, easy-to-use, compact and low-weight heavy metal analyzer combined with a disposable COC lab-on-chip devices is presented in [117]. The system includes seven disposable lab chips, a plastic fluidic motherboard with a microchannels network, microvalves and pump, control circuits, a wireless communication module, a potentiostatic battery and LabVIEW control, allowing low power consumption and automatic control. The proposed system was tested for the detection of Cd^2+^ using an electrochemical detection method based on a microfabricated planar bismuth electrode on the chip, while the system was also tested for the on-site detection of Cd^2+^ in soil pore and ground water samples. A. Jang et al. [118] have also integrated an electrochemical sensor in a polymer lab chip for on-site measurement of Cd^2+^. Using a bismuth electrode, which is an environmentally friendly solution, they detected 9.3 μg L^−1^ of Cd^2+^ in aqueous samples. They have also shown that, in a binary mixture of Cd^2+^ and Pb^2+^, Pb^2+^ will probably not significantly affect Cd^2+^ measurement in typical samples of interest (Figure 11A). In another effort, a portable and low-cost microfluidic chip with a miniaturized Ag/AgCl reference electrode for Cd^2+^ and Pb^2+^ in sea water is described in [119]. The detection limits of this device, which is again a PDMS microfluidic channel, are 0.34 and 0.15 ppm for Cd^2+^ and Pb^2+^ ions, respectively. A microfluidic device consisting of PDMS microfluidic channels and inkjet printed interdigitated electrodes made from silver on a flexible PET substrate is reported in [120]. For the formation of the final device, the PDMS and the PET substrate are bonded together. The proposed device is used for the detection of mercury sulfide and cadmium sulfide with concentrations down to picomolar levels. Y. Yuan et al. [121] have achieved the detection of cadmium ions, with a concentration down to 0.03 μg L^−1^, using a PMMA-based microfluidic electrochemical sensing platform based on GO/MWCNTs/Nafion modified SPEs.

In another example [122], filter paper strips are integrated with commercial SPCE for the fabrication of a paper-based microfluidic device. Thus, a portable and cheap electrochemical device is fabricated for the measurement of Pb^2+^ and Cd^2+^ in aqueous samples. A remarkable advantage of the device is that there is no need for a pretreatment step. With this device, concentrations down to 2.0 and 2.3 ppb for Pb^2+^ and Cd^2+^, respectively, are measured. Finally, a portable microfluidic platform with a microcantilever-based piezoresistive sensor was fabricated for Cd^2+^ detection in ground water (Figure 11B) [123]. The selective detection of the metal ions is performed through the use of a cysteamine-functionalized microcantilever-based sensor with cross-linked dl-glyceraldehyde. Thus, an ultra-sensitive detection of Cd^2+^ is achieved with a concentration down to 2.78 pM, which is below the WHO limit of 3 μg L^−1^, in a very short time (20–23 min).

### 2.5. Chromium (Cr) Detection

Chromium (Cr) is used in electroplating, chrome plating, leather tanning, dye and pigment production, wood preservation and metallurgy. It can further be found in water, sediments, soils, rocks, biota and volcanic emissions. Trivalent (Cr (III)) and hexavalent (Cr (VI)) chromium are the most stable forms of chromium in aqueous solutions. Although Cr (III) is essential for living organisms in a high dose, it can cause digestive problems and affect the kidneys and liver. Furthermore, Cr (VI) is considered highly toxic and carcinogenic for humans. These kinds of metal ions are probably released in the environment through waste water due to industrial activities but also from other human activities (i.e., agriculture activities). Drinking water is also polluted by Cr (III) and Cr (VI) as a result of the utilization of corrosion inhibitors in water pipes and containers or by the pollution of underground water [124,125,126]. Thus, the detection of chromium in water samples is necessary and, in this direction microfluidic devices with optical and electrochemical methods are widely used [125,127,128,129,130,131,132]. In the following paragraphs, we will discuss several attempts to detect Cr reported to date.

A simple glass microfluidic device with T channels based on CL is proposed for the detection of Cr (III) and total chromium monitoring in water samples [133]. The detection of Cr is relying on luminol oxidation via hydrogen peroxide in a basic aqueous solution catalyzed by Cr (III). In this way, Cr (III) with a concentration down to 1.6 × 10^−16^ mol L^−1^ is detected, while total Cr can be defined using the same strategy as Cr (III). W. Alahmad et al. [134] have also detected Cr (III) with a miniaturized chemiluminescence detection system, in which luminol is oxidized by hydrogen peroxide in the presence of Cr (III). In this case, the microfluidic device is fabricated on a paper substrate and it has six separate channels in a parallel alignment. Using this device, in which each channel has an injection zone for a reagent solution, a reaction zone and a waste zone, they have managed to detect Cr (III) in a very short time with an LOD of 0.02 ppm. Another paper-based microfluidic device using the same scheme as previously for Cr (III) detection is described in [135]. The main difference with other devices is that in this work a solely gravity and capillary force-driven flow chemiluminescence platform is fabricated. Using this low-cost, simple, fast and portable device, a concentration down to 0.0245 mg L^−1^ is measured in less than 30 s. H. Wang et al. [136] have also reported a paper-based microfluidic device for the colorimetric detection of various heavy metals in water samples. In particular, they achieved the colorimetric detection of Cd^2+^ and Cr (VI) with concentrations down to 0.19 ppm and 0.35 ppm, respectively. The color change is detected by imagining the metal chromogenic signals using a mobile phone camera and further analyzing the signal with image processing and analysis software. A paper-based microfluidic device in which the connection or disconnection among detection zones and fluid channels is controlled by rotational valves is described in [137]. In this way, cheap and portable devices for on-site measurement can be developed, while the particular device is used for the detection of various metals including Cr (VI). Using the appropriate colorimetric reagent (1,5-diphenylcarbazide), a concentration down to 0.18 mg L^−1^ is measured. F. Li et al. [138] have also detected Cr (VI) using a 3D μPAD. In particular, the device consisted of two layers: one for pretreatment (top layer) and a second for the detection (bottom layer). An L-type circuitous flow route is designed for the transportation of the pretreated sample solution to the detection zones, avoiding the random diffusion of the chromogenic reagents. In this way, the colorimetric performance is improved and the color uniformity and the assay accuracy are enhanced. They also used a smartphone to capture the color images, and the reported LOD was 0.1 mg L^−1^.

Hexavalent chromium with a concentration of 3 µg L^−1^ is detected in water samples by another µPAD [139]. Diphenylcarbazide (DPC) is again used for the colorimetric detection of Cr (VI), while for the quantitative analysis a commercial desktop scanner with ImageJ software is used. Abdellah Muhammed et al. [126] have developed a simple, portable, and low-cost μPAD, in which Cr (VI) and Cr (III) can be detected. The device uses the left channels for the detection of Cr (VI) using 1,5-diphenylcarbazide and the right channels for the total Cr detection. Using this colorimetric device, the limits of detection are 0.008 mg L^−1^ for Cr (VI) and 0.07 mg L^−1^ for Cr (III) or total Cr. In another effort reported in the literature, a PMMA microfluidic chip was coupled with a smartphone camera for Cr (III) detection [140]. The detection of Cr (III) is achieved through the color change in AgNPs, which are modified with pyrrolidine-1-dithiocarboxylic acid ammonium salt, upon Cr (III) exposure. The color change can be observed either by the naked eye or using a smartphone camera. Using a camera, they detected Cr (III) with an LOD of 9.18 nM. N. Mishra et al. [141] have fabricated a PDMS/glass-based microfluidic system for Pb^2+^, Cr (III) and Hg^2+^ detection in drinking water. In particular, the detection is achieved via the immobilization of nanoparticles in the microfluidic system, while the microabsorbance method is used for detection. They also report that their device enables label-free detection of a minimum of 0.5 ppb for all the aforementioned heavy metal ions.

**Figure 12:**
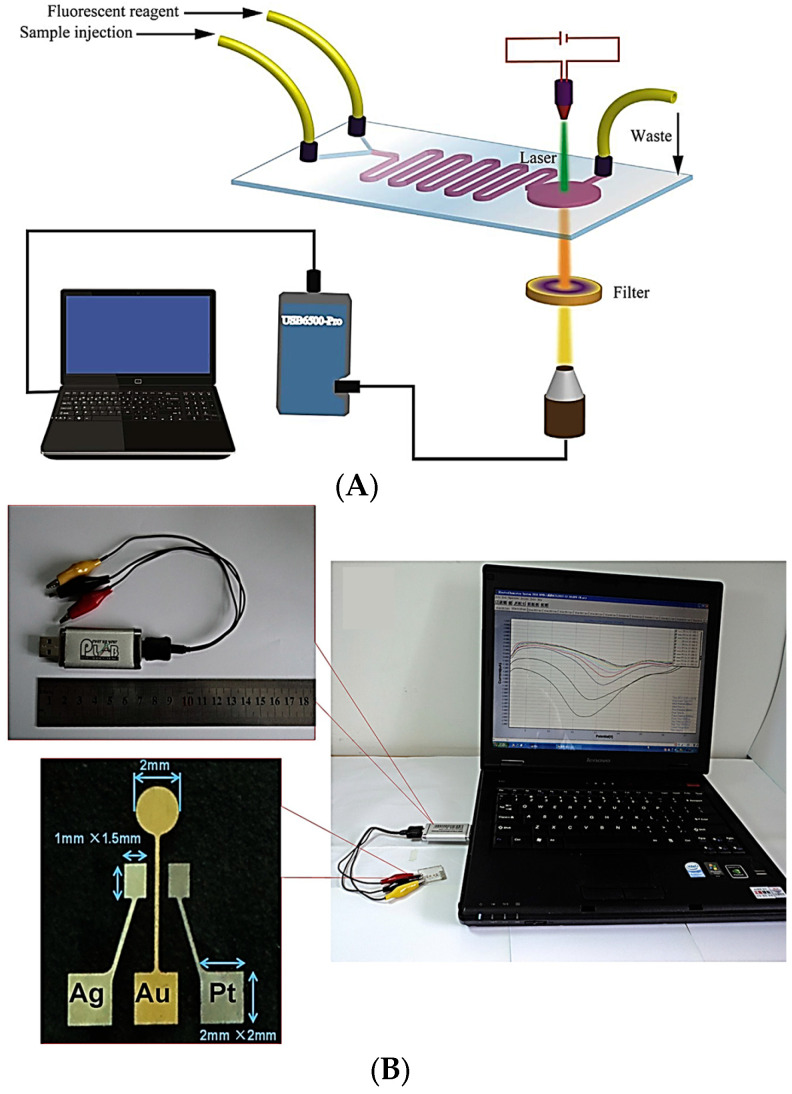
(**A**) Illustration of the experimental set up for the fluorescence detection of Cr (III) utilizing a PDMS microfluidic device. Reproduced with permission from reference [124]. (**B**) (**Right**) Image of the microchip sensor platform for Cr (VI) detection (including the PC for the readout), (**top left**) the USB electrochemical system and (**bottom left**) the sensor with the three electrodes (Au–Ag–Pt) on the PMMA substrate. Reproduced with permission from reference [131].

In another approach, RD is used for the detection of Cr (III) due to its high sensitivity and selectivity to Cr (III) and, thus, the fluorescence detection is achieved through the on-line derivatization of Cr (III) ions inside a PDMS microfluidic chip (Figure 12A) [124]. This chip has staggered herringbone grooves in the channel, allowing rapid and efficient mixing between the metal ions and the complex reagent, and it is combined with a portable laser-induced fluorescence detection system. With this method, which enables a low fluid volume consumption and low waste generation, this group measured concentrations down to 0.094 nM.

An easy-to-use and low-cost PMMA lab-on-disk, which is combined with an optical detection system, was presented for Cr (III) and Cr (VI) detection in water samples [142]. By combining the device with specific ligands, like 2,6-pyridine dicarboxylic acid for Cr (III) and DPC for Cr (VI), concentrations down to 21 mg L^−1^ and 4 μg L^−1^ for Cr (III) and Cr (VI), respectively, can be detected. M. Manachino et al. [143] tried to fabricate a device for autonomous and on-site measurement of heavy metals, such as Cr (VI). For this purpose, they have integrated microfluidic, electronical and optical devices via the miniaturization and optimization of the standard spectrophotometric analysis using specific ligands.

Compared to optical-based microfluidic devices for Cr detection, microfluidics devices with electrochemical sensors are limited. For example, a novel paper-based microfluidic device where colorimetric and electrochemical detection methods are used in a single device is described in [132]. In this case, separate detection layers, which enable the application of different chemistries, are used. Thus, the colorimetric detection method is used for the detection of Cr with an LOD of 0.12 μg and electrochemical detection method for Pb and Cd with an LOD of 0.25 ng. Y. An et al. [125] have also combined electrochemical and colorimetric detection in the same paper-based analytical device for the detection of the total chromium (Cr) and Cr (VI) in water samples via one injection. In particular, the device consists of a fiberglass filter paper with a patterned microchannel, enabling the fluidic control, Cr (III) pretreatment in the middle region and colorimetric detection at one end of the microchannel, which is modified with chromogenic agents, and a custom-made three-electrode ectrochemical detection system at another end of the microchannel. In this way, a cheap and easy-to-use device with a low reagent consumption is developed with which concentrations down to 0.01 mg L^−1^ and 0.06 mg L^−1^ detected for Cr (VI) and total Cr, respectively. Finally, an LOC device was fabricated and proposed for Cr (VI) detection [131]. In the work, an electrochemical sensor with three electrodes (Au–Ag–Pt) fabricated on a PMMA substrate is combined with a PDMS microchannel (Figure 12B). In addition, a custom USB electrochemical system is used enabling the device’s use for on-site measurements, since it is compact and has a rapid response time, and the LOD of the device is 0.9 μM.

### 2.6. Discussion on Perspectives and Challenges

The WHO [144] has already pointed out the existing danger that heavy metals pose to human health and thus the need for a high water quality and clean and safe drinking water. According to our review on the progress of microfluidic and LOC devices implemented for the detection of some of the most dangerous heavy metal ions, it turns out that up to now several efforts have been made using a wide range of different materials for the fabrication of such devices. The most common materials used include paper, thermoplastics such as PMMA and COC, and elastomers, i.e., PDMS, whereas the detection methods mainly used are optical and electrochemical, due to the high sensitivity and simplicity they offer.

In Table 1, we present a summary of microfluidic or LOC devices used for heavy metal ions detection together with information about the materials used for the device fabrication, the detection method and the LOD achieved. The limit values in drinking water, according to WHO standards, are 10 μg L^−1^ (up to 50 μg L^−1^ in some countries) for As, 3 μg L^−1^ for Cd, 50 μg L^−1^ for Cr, 10 μg L^−1^ for Pb and 6 μg L^−1^ for Hg.

Table 1 summarizes several microfluidic or LOC example approaches for the detection of metal ions to date. In many of these examples, the reported LOD can meet or even exceed the WHO standards. However, to our knowledge most of these efforts have not been transformed to diagnostic products. The main reasons for this slow uptake by the industry of these devices are (a) The fabrication complexity, which makes chips harder to manufacture, while at the same time requiring more complex external components to control and readout the sensors, thus limiting their autonomous operation or their user-friendly operation; (b) Less complex devices (i.e., paper-based ones), as, although they are user-friendly, they usually use a colorimetric detection scheme, which without coupling with a camera device and analysis software cannot meet the LOD requirements by the legislation standards and thus their use is limited to screening purposes only; (c) The technological maturity is, in most cases, low and the reproducibility of the device performance is limited. Many of the reported efforts have yet to be evaluated with complex real water samples and in relevant industrial environments; (d) Industry is generally reluctant to adapt new technologies, because time and investments are required to develop and then adopt the new approaches. To address these limitations, new fabrication methods, improvements in the detection schemes as well as in the devices required for the output of the results, and extensive evaluation protocols using real samples are required.

The food and water safety markets are highly regulated and, therefore, in order for new methods to proceed to the commercialization phase, specific legislations and directives should include them as accepted analysis methods. Otherwise, new methods will never be tested under real conditions because there will be no market interest. A representative example in order to better understand the reason for the slow uptake of new methods in the food and water safety market work is the following: Regarding the quality of water intended for human consumption, only recently the EU Directive [146] has included new detection methods (i.e., PCR-based methods) as alternatives to the gold standard method (plating) in microbiology analysis. It is therefore evident that new methods and devices can reach the commercialization phase only through close collaboration between different shareholders in the field of food and water safety (scientific community, public bodies, governments, non-governmental organizations, industry, accredited water safety companies and laboratories).

During the pandemic, in an effort to limit the spread of the SARS-CoV-2 virus, several methods were rapidly developed and accredited, thus opening a favorable environment in the post-COVID era for new methods and innovative devices to find their way to the health, food and water safety markets.

## 3. Conclusions

One of the scientific sectors in which microfluidic and LOC devices are widely used is that of environmental monitoring. One of the most important targets in environmental monitoring is water pollution by heavy metal ions as they can easily pass to drinking water. Miniaturized systems that take advantage of microfluidic devices equipped with appropriate detection schemes seem to be a very promising tools for water safety and the detection of heavy metals. The main reason is that such devices can offer sensitivity, specificity, portability and multiplexing in terms of multiple targets’ detection in a single device, specifications which are essential in water and environmental monitoring.

This review paper reports the most common microfluidic-based devices with a particular focus on the LOC approaches together with details on their fabrication methods, the materials used, their detection schemes and their performance capabilities. It is clear that state-of-the-art examples, which can meet the legislation limit values, have already been reported. However, despite this great progress in the field, several challenges still exist. According to our point of view, one of the biggest is the device integration, i.e., the incorporation of all the necessary parts (i.e., microfluidic channels, the detection unit, heating elements, electrical connections, etc.). Additionally, on their way towards possible commercialization, performance and detection repeatability is another aspect which could be improved along with an extensive industrially relevant evaluation of these devices, which is also missing. It is therefore evident that, further to the technology advancements that are required, it is also important to develop collaboration frameworks to address these shortcomings. In this way, important feedback can be provided by the end users, which can help the research community to work on the drawbacks and improve the performance and repeatability of LOC devices. To date, conditions are in favor of new technologies and methods and it is expected that during the next years more and more LOC and microfluidic devices in general will reach the commercialization level.

## Figures and Tables

**Table 1:** Summary of the most promising microfluidic or LOC devices categorized according to the heavy metal ions detected. The detection method, its LOD and the material used for the fabrication of each device are provided.

Heavy Metal Ion	Reported LOD	Device Material	Detection Method	Reference
**Single heavy metal ion detection devices**				
**Pb (II)**	5 μg L^−1^	PDMS and glas	Fluorescence	[57]
	1.2 nM and 0.7 nM, respectively, for each type of material	Whatman No.1 and nylon	Colorimetric using image analysis	[62]
	0.13 μg L^−1^	Glass–silicon–glass	Electrochemical	[77]
	40 ng L^−1^	NOA 81 polymer and silicon	Electrochemical	[145]
**Hg (II)**	0.031 μM	PDMS and glass	Fluorescence using an optical spectrometer	[83]
	1.4 μg L^−1^	Glass	Fluorescence with a CCD camera	[84]
	0.056 μg L^−1^	Paper	Fluorescence	[87]
	3 μg L^−1^	Paper	Colorimetric using a smartphone	[91]
	2.7 pM	PDMS and glass	LSPR coupled with a dark-field testing microfluidic platform	[97]
	3 μg L^−1^	PDMS	Electrochemical	[98]
	2.4 nM	PDMS	Electrochemical	[99]
**As (III)**	1.0 μg L^−1^	Paper	Colorimetric	[103]
	5 μg L^−1^	Paper	Colorimetric using a mini microscope	[105]
	0.67 μg L^−1^	PDMS	SERS	[107]
	0.42 μg L^−1^	PC, PMMA, PDMS	Electrochemical	[111]
**Cd (II)**	0.45 μg L^−1^	PDMS and glass	Fluorescence	[114]
	38.9 ng L^−1^	PDMS	Optical	[116]
	9.3 μg L^−1^	COC polymer	Electrochemical	[118]
	0.03 μg L^−1^	PMMA	Electrochemical	[121]
**Cr (III)**	0.094 nM	PDMS	Fluorescence using a portable fluorescence detection device	[124]
	24.5 μg L^−1^	Paper	CL coupled with image analysis	[135]
	9.18 nM	PMMA	Colorimetric using a smartphone coupled with image analysis software	[140]
**Cr (VI)**	4 μg L^−1^	PMMA	Optical	[142]
	10 μg L^−1^	Paper	Electrochemical	[125]
	0.9 μM	PMMA and PDMS	Electrochemical	[131]
**Multiple heavy metal ions detection devices**				
**Pb (II)** **Hg (II)**	0.53 μg L^−1^ and 0.70 μg L^−1^, respectively	PDMS	Fluorescence	[59]
	0.07 μg L^−1^ and 0.18 μg L^−1^, respectively	Cloth and paper	Fluorescence	[65]
	10 pM and 0.2 nM, respectively	Paper	ECL	[61]
**Pb (II)** **Cd (II)**	0.335 μg L^−1^ and 0.245 μg L^−1^	Paper	Fluorescence using a custom device and a smartphone	[64]
	1.8 μg L^−1^ and 1.2 μg L^−1^, respectively	Paper	Electrochemical	[68]
	1 μg L^−1^ and 25 μg L^−1^, respectively	Paper	Electrochemical	[69]
	2.0 μg L^−1^ and 2.3 μg L^−1^, respectively	Paper	Electrochemical	[122]
**Pb (II)** **As (III)** **Cd (II)**	4.44 nM, 5.03 nM and 41.1 nM, respectively	PDMS and glass	Fluorescence	[115]
	1.2 μg L^−1^, 2.4 μg L^−1^ and 0.8 μg L^−1^, respectively	3D-printed polymer flow cell from clear resin	Electrochemical	[113]
**As (III)** **As (V)**	1 μg L^−1^	Poly-urethane, flexible polymer film (PET/EVOH/PE) and FR-4	Colorimetric using a portable device equipped with a camera for image analysis	[106]
**Cr (III)** **Cr (VI)**	70 μg L^−1^ and 8 μg L^−1^, respectively	Paper	Colorimetric using image analysis and inductively coupled plasma-optical emission spectrometry	[126]
**Pb (II)** **Cr (III)** **Hg (II)**	0.5 μg L^−1^	PDMS and glass	Microabsorbance	[141]

## Data Availability

No new data were created. This review paper is focused on microfluidic-based and LOC devices used for the detection of specific heavy metal ions (Pb, Hg, As, Cd and Cr). Reports about other heavy metal ions are not included. In addition, the main timeframe was the last 6 years; however, highly cited papers from previous years are also included. The literature search was performed using Scopus and Google Scholar databases.
